# Clinical management and risk reduction in women with low-grade squamous intraepithelial lesion cytology: A population-based cohort study

**DOI:** 10.1371/journal.pone.0188203

**Published:** 2017-12-28

**Authors:** Yi-Jou Tai, Yun-Yuan Chen, Huang-Cheng Hsu, Chun-Ju Chiang, San-Lin You, Hui-Chi Chen, Chi-An Chen, Wen-Fang Cheng, Taiwan Cervical Cancer Control Task Force

**Affiliations:** 1 Department of Obstetrics and Gynecology, College of Medicine, National Taiwan University, Taipei, Taiwan; 2 Graduate Institute of Clinical Medicine, College of Medicine, National Taiwan University, Taipei, Taiwan; 3 Taiwan Blood Services Foundation, Taipei, Taiwan; 4 Graduate Institute of Epidemiology and Preventive Medicine, College of Public Health, National Taiwan University, Taipei, Taiwan; 5 Taiwan Cancer Registry, Taipei, Taiwan; 6 Department of Public Health, College of Medicine and Big Data Research Centre, Fu-Jen Catholic University, New Taipei City, Taiwan; 7 Graduate Institute of Oncology, College of Medicine, National Taiwan University, Taipei, Taiwan; Rudjer Boskovic Institute, CROATIA

## Abstract

We analyzed the management and risk of subsequent cervical intraepithelial neoplasm 3 (CIN3) and invasive cervical cancer in women with low-grade squamous intraepithelial lesion (LSIL) cytology. A total of 53,293 women with a new diagnosis of cytologic LSIL were identified in Taiwan’s national cervical screening registration database. Based on the retrieved clinical management data, the incidence of subsequent CIN3+ lesions was determined, and the hazard ratios (HRs) were estimated using a Cox proportional hazards model. The average follow-up was 5.02 years. A total of 988 women developed CIN3+ lesions during this period, with an overall incidence of 369.3 women per 100,000 person-years. Cryotherapy and conization/loop electrosurgical excision procedure (LEEP) decreased the subsequent risk of CIN3+ lesions in women younger than 50 years (HR 0.49, 95% confidence interval [CI] 0.37–0.64, p<0.0001 for cryotherapy; HR 0.39, 95% CI 0.27–0.55, p<0.0001 for LEEP). Cryotherapy and conization/LEEP were two significant protective factors for developing CIN3+ lesions, especially in women with biopsy-proven CIN1 (HR 0.55, 95% CI 0.37–0.82, p = 0.003 for cryotherapy; HR 0.43, 95% CI 0.24–0.77, p = 0.005 for LEEP). These results suggest that when women are first screened LSIL and lack prior abnormal cervical cytology, cryotherapy should be one of the treatment options. Younger women with a histological biopsy diagnosis of CIN1 were most likely to benefit from cryotherapy.

## Introduction

Cervical cancer is the third most common cancer in women, with an estimated 528,000 new cases and 266,000 deaths worldwide per year. [1] According to the 2013 annual cancer registry report in Taiwan, the incidence of cervical cancer is the seventh most common cancer in women, and a total of 1,579 women were diagnosed in 2013, giving an age-adjusted incidence of 9.46 per 100,000 person-years. Pap smears are performed to obtain cells from the cervix and vagina for the detection of cervical cancer and its precursors. Pap smears can be an effective tool for reducing the incidence of cervical cancer and the associated mortality. For example, a cervical cancer screening program was launched in Taiwan in 1995 that included annual Pap test reimbursement; this decreased the incidence of invasive cancer by 47.8% in 1995–2006. [2] In Taiwan, cervical screening using either Pap smear or liquid based cytology starts three years after a woman becomes sexually active or when she is 30 years old, and there is no upper age limit for screening. The recommended screening interval is one year, although it can be modified to every three years after three consecutive negative screenings. At present, the screening program in Taiwan does not use combined cytology and human papillomavirus (HPV) testing. Women aged 20–29 years who are not covered by the national screening program can still participate in screening if they have risk factors such as early commencement of sexual activity, a high number of partners, or a history of abnormal bleeding. HPV testing is not recommended in women aged < 30 years.

Low-grade squamous intraepithelial lesion (LSIL) was first introduced in the 2001 Bethesda system. [1] LSIL is defined as the cytopathic effect of transient HPV infection known as koilocytosis and mild dysplasia or cervical intraepithelial neoplasia 1 (CIN1). [3] The median duration of HPV infection is 8 months, and persistent infection increases the risk of developing or the persistence of squamous intraepithelial lesions.[4] The reporting rates for LSIL range from 1.6% to 7.7% in different countries.[5–7]

The majority of women with LSIL test positive for HPV, with 90% of women showing spontaneous clearance within 2 years, especially adolescents and young women.[8] Low-grade lesions frequently regress; therefore, repeating the cytology provides time for regression to occur.[9] The practice guidelines for the management of LSIL in Taiwan is colposcopic exam, except in adolescents. Adolescents are managed with follow-up cytology at 3- to 6-month intervals, and colposcopy is recommended when cytology shows atypical squamous cells or greater lesions.

The American Society for Colposcopy and Cervical Pathology (ASCCP) guidelines recommend that women with cervical cytology that is interpreted as LSIL be managed by immediate colposcopy,[10, 11] except for adolescents. When HPV co-testing is available, it is preferred that women with HPV-negative LSIL repeat the co-testing one year later, but colposcopy is also acceptable. Follow-up with annual cytological testing is recommended thereafter. According to the atypical squamous cells of undetermined significance (ASCUS)-LSIL Triage Study (ALTS), the cumulative risk of CIN2 or CIN3 at two years is 27.6% for LSIL, and initial colposcopic detection of obvious CIN2 or CIN3 can reduce this risk.[12] Few studies have investigated the management of LSIL cytology without histological LSIL. Notably, the influence of different treatment options, such as ablation or excision, on the development of more serious lesions has not been well studied. Accordingly, we used data from Taiwan’s national cervical cancer screening database to evaluate the impact of different management modalities on the outcomes of women with LSIL cytology who subsequently developed CIN3+ lesions.

## Materials and methods

**Fig 1:**
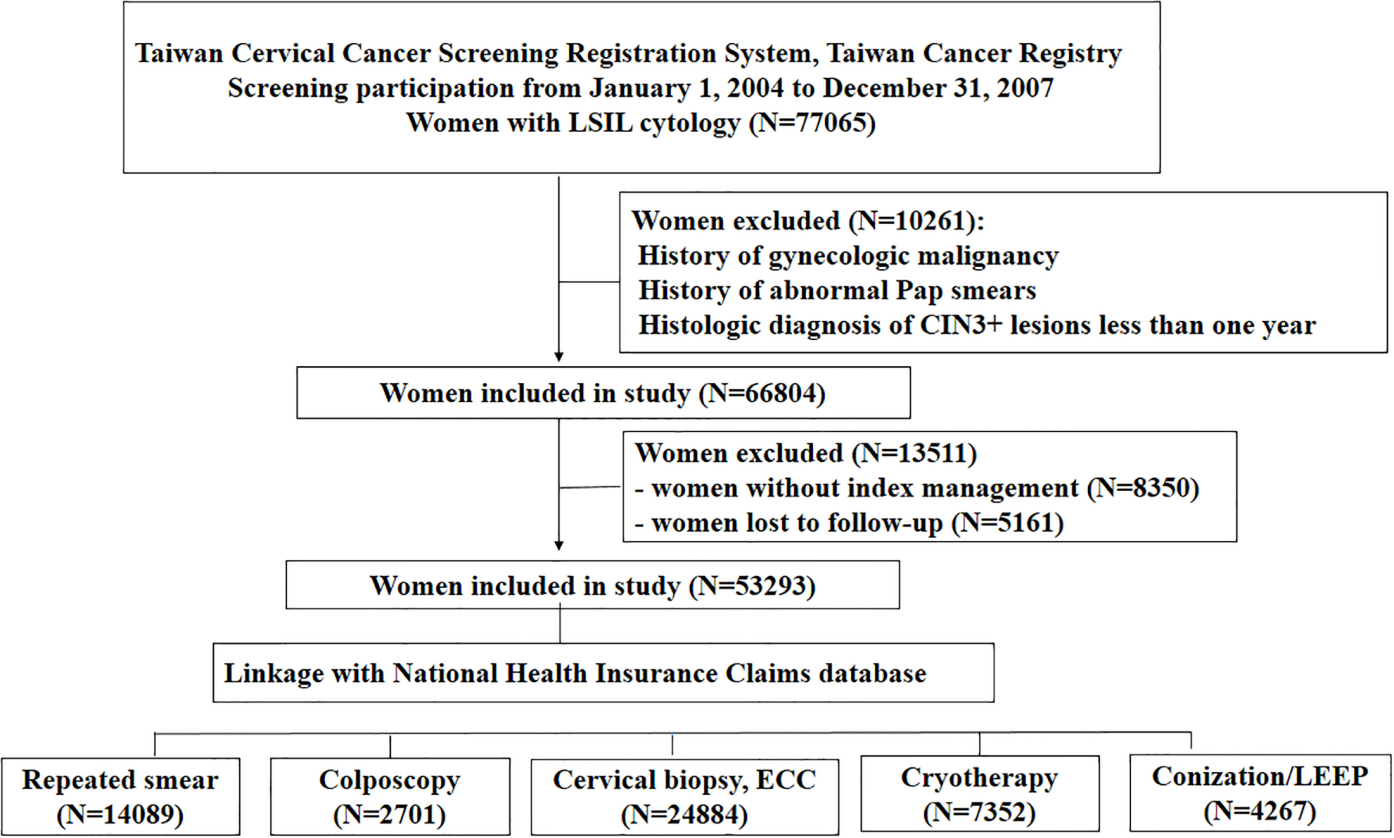
Flow chart of the study population, which included all women with LSIL cytology who were screened in 2004–2007 in Taiwan.

This study was approved by the Research Ethics Committee of the National Taiwan University Hospital and was registered at ClinicalTrials.gov (identifier NCT02063152). The records of women with LSIL cytology from January 1, 2004 to December 31, 2007 were retrieved from the following databases: the Taiwan Cervical Cancer Screening Registration System, Taiwan Cancer Registry, and the National Health Insurance Claims Database. The retrieved information included personal identification information, date of birth, date of diagnosis, histological diagnosis, and treatment (Fig 1). The exclusion criteria were gynecologic malignancy (malignancies involving the uterine corpus, cervix, ovary, or vagina) and a history of abnormal Pap smears (including CIN, atypical cells, or malignant cells) before the detection of LSIL cytology. Women were also excluded if the interval between the LSIL cytology finding and the histologic diagnosis of CIN3+ lesions was less than one year in order to avoid the inclusion of occult CIN3+ lesions from the initial screenings. Eligible women with LSIL cytology were followed up to the time of the CIN3+ lesion, including the CIN3 diagnosis and invasive cervical cancer diagnosis. The follow-up period was one year after the LSIL cytology results to the time that a CIN3+ lesion was diagnosed, the date of death, or December 31, 2010, whichever came first. Information about clinical management that was performed within one year after detection of LSIL cytology (irrespective of management one year later) was retrieved from the National Health Insurance Claims Database and included information about repeated Pap smears, colposcopy without biopsy, cervical biopsy, endocervical curettage (ECC), and interventional procedures (cryotherapy of the cervix, LEEP, or conization). If women with LSIL cytology had multiple disease management procedures, the more invasive management modality was used for the analysis. For example, if a colposcopy, cervical biopsy, and/or ECC were performed, the latter was used as the index management. If a cervical biopsy and LEEP/conization were both performed, the latter was regarded as the index management.

The incidence and hazard ratios (HRs) were estimated in order to evaluate the risk of subsequent CIN3+ lesions according to different types of disease management. The incidence of CIN3+ lesions was calculated by dividing the number of cases with CIN3+ lesions by the number of person-years at risk of developing CIN3+ lesions. Cox proportional hazards regression analysis was used to estimate the HRs with 95% confidence intervals (CIs), which were adjusted for age, educational status, residency, and previous screening interval. The cumulative risk of subsequent CIN3^+^ in women with first-time cytologic LSIL of the cervix was determined using Kaplan-Meier survival analysis and the log-rank test. Statistical significance levels were determined using a two-tailed test, and a p value <0.05 was considered statistically significant. Statistical analyses were performed using SAS software (version 9.3; SAS, Inc., Cary, NC).

## Results

A total of 53,293 women were identified who were newly diagnosed with LSIL cytology from January 1, 2004 to December 31, 2007. There were a total of 267,490.7 person-years of follow-up, with an average follow-up time of 5.02 years. During this period, CIN3+ lesions were diagnosed in 988 women, and the overall incidence of CIN3+ lesions was 369.3 per 100,000 person-years. Table 1 shows the subsequent incidence of CIN3+ lesions in these 53,293 women according to age, previous Pap test interval, and type of treatment management. We found that 10.6% of the women younger than 30 years and 10.5% of the women older than 60 years developed subsequent CIN3+ lesions; 67.8% of the women were 30–49 years old. In these 53,293 women, 23.8% had not had a Pap test in the previous 5 years or were never screened, whereas 21.6% had been screened in the previous year. Regarding treatment, 87.2% received non-interventional procedures, such as repeated Pap smears, colposcopy, and cervical biopsy and/or ECC. The remaining 12.8% had interventional procedures such as cryotherapy, conization, or LEEP.

**Table 1:** Baseline characteristics of the 53,293 women with LSIL cytology in this study and the management and incidence of subsequent CIN3+ lesions.

	LSIL patients n (%)	Person-years of follow-up	Cases with CIN3+ lesions n (%)	Incidence of CIN3+ lesions per 100,000 person-years*
**Age, years**				
<30	7,513 (14.1)	37,151.8	105 (10.6)	282.6
30**–**39	18,218 (34.2)	91,144.7	377 (38.2)	413.6
40**–**49	16,507 (31.0)	84,251.7	292 (29.6)	346.5
50**–**59	7,271 (13.6)	35,190.9	110 (11.1)	312.5
60**–**69	2,673 (5.0)	14,161.7	75 (7.6)	529.5
>70	1,111 (2.1)	5,589.7	29 (2.9)	518.8
**Previous screening interval**				
<1 year	11,859 (22.2)	63,290.1	213 (21.6)	336.5
1–3 years	22,971 (43.1)	114,425.0	427 (43.2)	373.1
3–5 years	5,218 (9.8)	24,318.8	113 (11.4)	464.6
>5 years or never screened	13,245 (24.9)	65,456.8	235 (23.8)	359.0
**Management**				
Repeated smear	14,089 (26.4)	74,811.9	304 (30.8)	406.3
Colposcopy	2,701 (5.1)	13,407.6	47 (4.8)	350.5
Cervical biopsy and/or ECC	24,884 (46.7)	117,220.7	510 (51.6)	435.0
Cryotherapy	7,352 (13.8)	35,574.7	80 (8.1)	224.8
Conization/LEEP	4,267 (8.0)	26,475.6	47 (4.7)	177.5
**Total**	53,293 (100)	267,490.7	988 (100)	369.3

Abbreviations: LSIL = low-grade squamous intraepithelial lesion, ECC = endocervical curettage,

LEEP = loop electrosurgical excision procedure.

* Age-adjusted incidence rate of CIN3+ lesions per 100,000 person-years

### Multivariate analysis identified age and management as independent risk factors for subsequent CIN3+ lesions in 53,293 women with LSIL cytology

Table 2 shows the risk factors for the development of CIN3+ lesions. Compared to women aged 30 to 39 years, women aged younger than 30 years (HR 0.67, 95% CI 0.54–0.84, *p* = 0.0005), 40–49 years (HR 0.81, 95% CI 0.69–0.95, *p* = 0.009), and 50–59 years (HR 0.71, 95% CI 0.56–0.90, *p* = 0.005) had lower risks of developing CIN3+ lesions by multivariate analysis. The screening intervals between the previous normal and the current LSIL Pap test did not correlate with the risk of developing CIN3+ lesions. Women with LSIL cytology who underwent interventional procedures, including cryotherapy (HR 0.55, 95% CI 0.43–0.70, *p*<0.0001) or conization/LEEP (HR 0.45, 95% CI 0.33–0.61, *p*<0.0001), had a significantly lower risk of developing CIN3+ lesions compared with the reference group of women who only received repeated smears.

**Table 2:** Multivariate Cox proportional hazard model analysis of the risk of subsequent CIN3+ lesions in 53,293 women with LSIL cytology.

	HR	95% CI	p value
**Age, years**			
<30	0.67	0.54–0.84	0.0005
30–39	1		
40–49	0.81	0.69–0.95	0.009
50–59	0.71	0.56–0.90	0.005
60–69	1.21	0.90–1.62	0.22
>70	1.14	0.75–1.73	0.54
**Previous screening interval**			
<1 year	0.92	0.78–1.08	0.29
1–3 years	1		
3–5 years	1.19	0.97–1.47	0.09
>5 years or never screened	1.03	0.87–1.22	0.71
**Management**			
Repeated Pap smear	1		
Colposcopy	0.89	0.65–1.21	0.44
Cervical biopsy and/or ECC	1.07	0.92–1.23	0.38
Cryotherapy	0.55	0.43–0.70	<0.0001
Conization/LEEP	0.45	0.33–0.61	<0.0001

HR: hazard ratio, CI: confidence interval

Age, previous screening interval, and management were included in the adjusted analysis.

The risk of subsequent CIN3+ lesions was lower in younger women, women between 40 and 59 years old, and women who received an intervention after being screened for LSIL cytology.

### Multivariate analysis to identify risk factors for subsequent CIN3+ lesions in 53,293 women with LSIL cytology, with or without CIN1 pathology

Women with LSIL cytology were analyzed based on their cervical biopsy pathology results, which were divided into two groups: group 1 had no CIN pathology (normal results, atrophy, or inflammation), and group 2 had CIN1 pathology. As shown in Table 3, women with LSIL cytology but without CIN pathology did not have a lower risk of developing CIN3+ lesions than women who only had Pap smears, regardless of whether they received colposcopy, cervical biopsy/LEEP, cryotherapy, or conization/LEEP (all *p* values > 0.05). Women with LSIL cytology who had CIN1 pathology and were treated with cryotherapy (HR 0.55, 95% CI 0.37–0.82, *p* = 0.003) or LEEP/conization (HR 0.43, 95% CI 0.24–0.77, *p* = 0.005) had a significantly lower risk of subsequent CIN3+ lesions than women who only had Pap smears. This was not observed in women with LSIL cytology without CIN pathology.

**Table 3:** Multivariate analysis of the risk for subsequent CIN3+ lesions after adjusting for age and screening interval in 53,293 women with LSIL cytology and/or CIN1 pathology.

	Cases, n	HR	95% CI	p value
Repeated smear	304	1		
Colposcopy	47	0.88	0.65–1.20	0.43
Cervical biopsy and/or ECC				
No CIN pathology	121	0.98	0.79–1.21	0.83
CIN1	154	0.99	0.81–1.21	0.92
Cryotherapy				
No CIN pathology	7	0.39	0.19–0.83	0.02
Cervical biopsy: CIN1	27	0.55	0.37–0.82	0.003
Conization/LEEP				
No CIN pathology	6	0.62	0.27–1.38	0.24
CIN1 pathology	12	0.43	0.24–0.77	0.005

HR: hazard ratio, CI: confidence interval

Our results indicated that cryotherapy and conization/LEEP are two strong protectors against subsequent CIN3+ lesions in women with both LSIL cytology and CIN1 pathology.

### Age and disease management as risk factors for subsequent CIN3+ lesions in 53,293 women with LSIL cytology

We also evaluated the influence of age and type of disease management on women with LSIL cytology. Women with LSIL cytology were divided into 5 groups according to their age and type of disease management. As shown in Table 4, there was no difference in the HR of developing subsequent CIN3+ lesions in women who were younger versus older than 50 years old when their disease was managed with repeated Pap smears, colposcopy, cervical biopsy, and/or ECC compared to woman younger than 50 years old who only had repeated Pap smears. Cryotherapy (HR 0.49, 95% C.I. 0.37–0.64, *p*<0.0001) and conization/LEEP (HR 0.39, 95% C.I. 0.27–0.55, *p*<0.0001) lowered the HR of developing subsequent CIN3+ lesions in women younger than 50 years old but not in women aged older than 50 years compared to the reference group that was treated only with Pap smears.

**Table 4:** Multivariate analysis of the risk of developing subsequent CIN3+ lesions according to age and disease management in 53,293 women with LSIL cytology.

Treatment and age group	HR	95% CI of HR	p value
**Repeated smears**			
≤50 years	1		
>50 years	0.72	0.42–1.23	0.23
**Colposcopy**			
≤50 years	0.85	0.61–1.20	0.36
>50 years	0.74	0.34–1.62	0.45
**Cervical biopsy and/or ECC**			
≤50 years	1.03	0.87–1.20	0.77
>50 years	0.90	0.54–1.5	0.69
**Cryotherapy**			
≤50 years	0.49	0.37–0.64	<0.0001
>50 years	0.68	0.35–1.32	0.25
**Conization/LEEP**			
≤50 years	0.39	0.27–0.55	<0.0001
>50 years	0.54	0.26–1.13	0.10

HR: hazard ratio, CI: confidence interval

Taken together, these results indicated that cryotherapy and conization/LEEP may protect women with LSIL cytology who are younger than 50 years old from developing CIN3+ lesions.

### The cumulative risk of CIN3+ lesions was significantly associated with disease management

**Fig 2:**
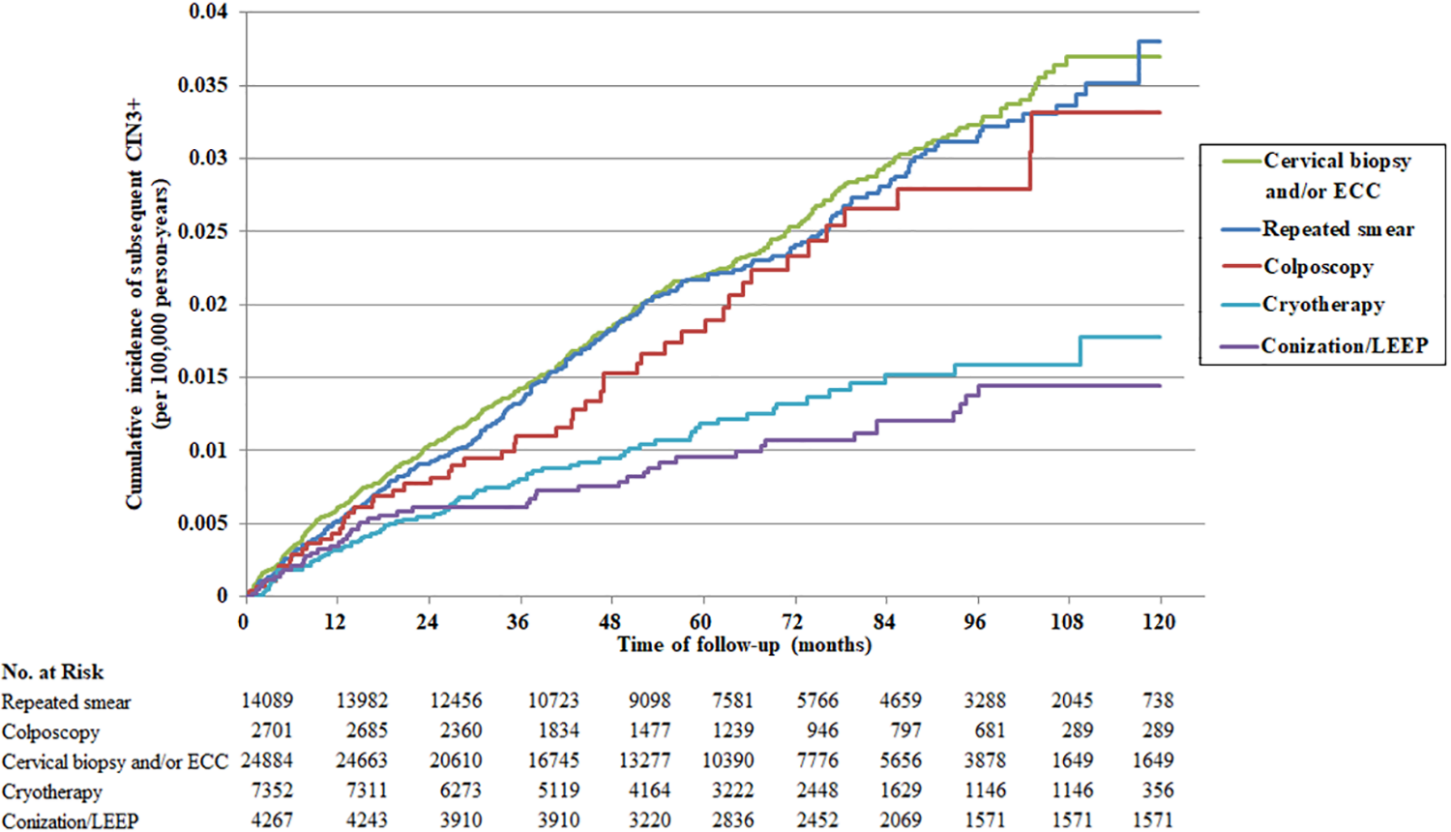
Kaplan–Meier curves for the risk of a subsequent CIN3+ diagnosis in 53,293 women with LSIL cytology according to disease management. The x-axis shows the follow-up time in months after the cytologic diagnosis of LSIL. The y-axis shows the cumulative incidence of subsequent CIN3+.

The cumulative risk of subsequent CIN3+ lesions in women with first-screened LSIL cytology was significantly different among the various management modalities (p<0.001, log-rank test) (Fig 2).

## Discussion

LSIL cytology reflects HPV infection. LSIL is compatible with a histologic diagnosis of CIN1, and the majority of LSIL cases spontaneously regress. In the Taiwan Cooperative Oncology Group (TGOG) study, the age-adjusted HPV prevalence was 12.6% in women with normal cytological findings and 74.9% in women with LSIL who were positive for HPV.[13] About 70% of CIN1 lesions spontaneously regress; this percentage is even higher, 90%, in adolescents and young women.[14, 15] The probability of progression to invasive cancer is approximately 1% in women with LSIL cytology,[16] and the risk of concomitant invasive cancer is rare. Untreated CIN1 lesions confer a risk of 12% for CIN2 or CIN3 at the two-year follow-up.[12] The decision to observe or treat women with LSIL cytology is always based on the individual’s risk. Current recommendations for managing women with LSIL cytology with no prior abnormal cervical screenings include a follow-up Pap smear or colposcopy.[11] HPV testing is advocated for women aged 30 years or older, and colposcopic examination is reserved for those who test positive for HPV.[17] Identifying women who are at a higher risk of progression to CIN2/3 lesions or invasive cancers and lowering their risk remains a challenge. Our series assessed the outcomes of women with LSIL cytology who were managed with different strategies to identify those who are at lower risk of developing CIN3+ lesions.

Age is related to the risk of developing CIN2+ lesions in women with LSIL cytology. In this study, younger women (<30 years old) and women aged 40–59 years had lower risks of developing CIN3+ lesions. Indeed, women younger than 30 years old had the lowest risk of developing CIN3+ lesions in our series. Reduced risk of CIN3+ lesions was also observed in women aged 40–49 and 50–59 years (Table 2). One possible explanation for these observation is the bimodal age-related peaks of HPV infection.[18, 19] The first peak occurs in young, sexually active women, with an age prevalence of 25 years, which may reflect transient HPV infection. The second minor peak occurs at older ages, mainly 45–50 years, and this peak may be due to the hormonal changes associated with menopause or changes in sexual behavior.

Prior screening history did not correlate with the risk of developing CIN3+ lesions in women with LSIL cytology in our study (Table 2). However, previous screening frequency was related to the development of invasive cervical cancer in women with ASC-US.[20, 21] The risk of developing cervical cancer was significantly higher in unscreened women and decreased with increasing screening frequency.[20] The HPV-positive rate in women with ASC-US was 50.6%, and this rate varied in different age groups.[22] It is possible that the high rates of HPV infection in women with LSIL cytology suggest that HPV infections can spontaneously resolve regardless of screening schedule.

Women with follow-up cytology testing had a similar risk of developing CIN3+ lesions as those treated with colposcopy alone or with cervical biopsy, suggesting that the spontaneous clearance of HPV during its natural history.[23] Cryotherapy and conization/LEEP could reduce the incidence of developing CIN3+ lesions in women with LSIL by improving the clearance of HPV infection. Our results showed that cryotherapy and excisional procedures, including conization or LEEP, substantially reduced the risk of developing CIN3+ lesions (Table 2). In particular, cryotherapy and conization/LEEP reduced this risk in women with LSIL cytology who had histologic CIN1 lesions (Table 3) and in women younger than 50 years old (Table 4). Elfgren et al. reported that the HPV clearance rate is 80% in women with LSIL who underwent cryotherapy and 95% in women with HSIL after LEEP.[24] A meta-analysis also showed that cryotherapy can achieve a 94% cure rate in women with CIN1.[25] Cryotherapy is an effective and safe procedure and is readily available for outpatient use. The adverse effects of cryotherapy are cramping and watery, serosanginous discharge with local cervical infection, although the latter is less frequent. Despite its effectiveness, cryotherapy is recommended for lesions that are limited to the ectocervix without endocervical extension when there is no concern that there is an underlying microinvasive cancer that might be misdiagnosed. Ablative surgery such as cryotherapy or conization/LEEP excisional procedures is thought to trigger local inflammation; this could serve to recruit immunocytes as part of the local immune response and thereby hasten HPV clearance and CIN regression. Colposcopy is mandatory for women with LSIL cytology women. ALTS reported that about one quarter (27.6%) of women with LSIL cytology developed CIN2+ lesions during 2 years of follow-up.[12] The colposcopic impression of CIN1 demonstrates 75.1% benign changes plus CIN1, while 25% of lesions are CIN2 or CIN3.[26] For women with initial LSIL cytology, “see and treat” using LEEP without biopsy-confirmed CIN2 or CIN3 is not recommended because of concerns of overtreatment.[27] The complications of LEEP include bleeding, infection, and impaired fertility, such as cervical stenosis, abortion, and preterm birth.[28]

One limitation of this study was the lack of data regarding HPV prevalence and the HPV type distribution for women with cytologic LSIL. HPV testing is not part of the recommended management of LSIL cervical cytology in the current practice guidelines in Taiwan, and it is not covered by National Health Insurance. Another limitation was that our study was based on a retrospective analysis of data from a nationwide screening database rather than being a randomized controlled study. Management was judged subjectively by clinicians and was based on the individual’s risk factors.

This study also has some strengths. The sample size in this population-based cohort study was sufficiently large, and the analysis was based on data from the National Health Insurance Claims database, which has a high coverage rate of cervical cytology screening. National Health Insurance in Taiwan covers 90%–98% of the general population, with reimbursement for annual conventional Pap smears for women over 30. This study directly compared the treatment effects and risks of different modalities and provided evidence that cryotherapy and excisional procedures reduce the subsequent risk of CIN3+ in women with a first diagnosis of LSIL cytology. Colposcopy is recommended for management of cytologic LSIL in Taiwan, and the treatment options for women with LSIL are either observation or ablative surgery. In light of the findings of our study, cryotherapy is an acceptable option and the treatment of choice, especially in young women with a satisfactory colposcopic examination, histologic CIN1, and in cases in which preservation of fertility is a concern.
